# Mask wearing provides psychological ease but does not affect facial expression intensity estimation

**DOI:** 10.1098/rsos.230653

**Published:** 2023-08-30

**Authors:** Toshikazu Kawagoe, Wataru Teramoto

**Affiliations:** ^1^ School of Humanities and Science, Tokai University, Kumamoto Campus, Toroku 9-1-1, Kumamoto City, Kumamoto 862-8652, Japan; ^2^ Division of Cognitive Psychology, Kumamoto University, 2-40-1 Kurokami, Chuo-ku, Kumamoto 860-8555, Japan

**Keywords:** face mask, facial expression, mask wearer, emotional judgement

## Abstract

During the COVID-19 pandemic, wearing a face mask became a global daily practice. Japanese people were already accustomed to wearing masks due to their collectivistic culture, which prioritizes conformity and group harmony. In such a culture, where individuals are concerned about how others perceive them and their actions, wearing masks can be a self-protective action to prevent, escape, or reduce the severity of perceived negative feelings. Previous studies indicate that people experiencing anxiety tend to have negative biases when evaluating emotional expressions on faces. Therefore, we hypothesized that wearing a mask can reduce the negative feelings caused by social pressure, emotion processing, especially intensity perception. While our findings confirmed that wearing a mask reduced negative feelings caused by social pressure, there was no significant change in emotion intensity recognition performance. This null result might be attributed to the small effect size of the association between negative bias in emotion processing and an individual's state. In future studies, it would be valuable to include participants from non-collectivistic cultures to gain a broader understanding of the impact of wearing masks on emotion processing.

## 1. Introduction

The COVID-19 pandemic originated in Wuhan, China, on November 17, 2019 [1], and rapidly spread globally, causing drastic changes that continue to impact society. While certain regions are experiencing an economic recovery, the pandemic's effects on mental health, including stress, anxiety, depression, frustration, and uncertainty, persist due to its ongoing progression, quarantine measures, and infections (for review, see [2]). In this time and age, one of the most characteristic daily practices is wearing a sanitary mask [3]. Although negative side effects have been reported [4], wearing a mask is generally considered a beneficial way to suppress the spread of infection [5,6].

In Asian countries, people do not hesitate to wear sanitary masks [7,8]. Japanese people wore masks even in the early stage of the COVID-19 pandemic [9]. This is because, in Japan, wearing masks has been ‘a unique and curious social common practice’ long before the COVID-19 pandemic [10–12]. Nevertheless, limited information exists on the impact of mask-wearing in Japan before the pandemic. For instance, Miyazaki & Kawahara [13] reported that attractive faces wearing sanitary masks were perceived as less attractive than without the mask (but see [14]).

After the pandemic, several global researchers have investigated the impact of mask-wearing on cognition, especially regarding face perception and emotion processing. Observers struggle to accurately perceive the facial expressions of mask wearers in terms of both valence and intensity due to mask interference [15–17], which can lead to miscommunication in clinical settings [18]. Conversely, mask-wearing also affects the wearer. For individuals with social anxiety, a mask acts as a ‘safety behaviour’, concealing the face and reducing the fear of negative evaluation [19]. Masks can be seen as barriers to social interaction, and those with social anxiety use them as safety measures to hide their face (for example, to hide blushing) [20]. Currently, people with social anxiety wear a mask not only to avoid contracting COVID-19 but also because of their self-concealing function (for review, [3]). The present study focuses on exploring the self-concealing function of face masks.

The prevalence of this ‘unique and curious social common practice’ [10] in Japan can be attributed to its collectivistic culture, which prioritizes conformity and harmony within groups over individualism. This cultural perspective assumes that individuals belong to one or more close ‘in-groups’ from which they cannot detach themselves [21]. In such a culture, exemplified by ‘taijin kyofusho’ (fear of interpersonal relations), a specific type of social anxiety [22], Japanese individuals tend to worry about how others evaluate them and their actions. In line with this, Nakayachi *et al*. [23] reported that social psychological motivations, rather than risk reduction expectations, adequately explain why Japanese people wear masks. Japanese individuals adhere to societal norms by wearing masks and experience relief from anxiety through this practice.

Thus, we quantitatively investigated the effect of wearing a mask on the wearer's psychological state and the cognition among Japanese people. To the best of our knowledge, only one study has investigated the effect of observers' mask-wearing on their cognition. Freud, di Giammarino, and Camilleri [24] systematically manipulated mask-wearing of both the observer and observed person to investigate whether mask-wearing affected face recognition ability. The results showed that irrespective of whether the observed person was masked or unmasked, face recognition was impaired when the observer wore a mask. This effect cannot be attributed to the congruency in wearing masks between the observer's and the observed person's face. Thus, an observer's state (i.e. wearing a mask or not) could substantially affect their face recognition ability. Additionally, this effect was absent when recognition targets were non-face objects, demonstrating the specificity of the mask-wearing effect to face processing. Although previous studies have reported that enforced face pose (e.g. biting a pen) or brain stimulation to the somatosensory areas could alter the face processing ability, they have also reported that the effect could be limited to emotion recognition but not to face perception [25,26]. Wearing a mask may provide more robust and consistent somatosensory stimulation for multiple face features compared to manipulating face pose or applying brain stimulation. Face pose manipulation (e.g. biting a pen) indirectly limits the mobility of specific muscles around the mouth, while brain stimulation only results in temporary changes in somatosensory perception [24]. Wearing a mask can have an equivalent or even stronger impact on emotion recognition compared to those modulations.

We hypothesized that (1) wearing a face mask reduces participants' negative feelings, such as threat, anxiety and nervousness; (2) observers wearing masks would perceive vague facial expression as less negative compared to when they do not wear masks. Negative feelings can disrupt emotion recognition, which is a fundamental human ability that provides a wide range of benefits in daily life. People with anxiety tend to exhibit negative bias when judging facial emotion expressions with lower intensity [27–29]. This, in turn, leads to increased detection of negative expressions [30,31]. Both trait and state anxiety can produce such effects, but state anxiety, which can be experimentally manipulated, holds more influence on emotion processing [32,33]. If wearing masks works as a safety behaviour or self-concealing tool, participants would feel more comfortable masked than unmasked. Consequently, the perceptual interpretation of less intense (or ambiguous) facial expressions of emotion may be less negatively biased, which can be captured behaviourally. In the present study, we investigated the influence of mask-wearing on the judgement of facial expressions of emotion by manipulating the participants’ state. Three different facial expressions (anger, fear and happiness) were used. We morphed each facial expression with neutral faces to create five levels of emotional intensity and measured the participants' sensitivity to facial emotion expressions via psychophysical techniques that could detect subtle conditional changes that the participants were not aware of [34]. Participants rated the intensity of facial expressions on six-point scales.

## 2. Methods

### 2.1. Participants

We conducted an *a priori* sample size calculation for the dependent sample *t*-test, considering a power value of 0.80, an effect size of 0.50, and a significance level of 0.05. The minimum sample size required was estimated to be 34 participants. However, we recruited 35 undergraduate students, considering potential errors, but none were excluded from the study (age = 20.3, 20 women). Participants were compensated for their time with 1000 Japanese yen. No participant was excluded due to cognitive, speech and language, and neurological impairments based on their self-reported information. All participants provided written informed consent to participate, and the study protocol was approved by the ethics review board at Kumamoto University.

### 2.2. Materials

An intensity rating task was conducted to assess face perception abilities. In this experiment, the independent variable was the mask condition (participants were masked or unmasked). Each trial presented a single face, which was created by morphing between a neutral face and a specific type of emotion (i.e. anger, fear, and happiness). To achieve this, we used a morphing software package, InterFace (https://www.york.ac.uk/psychology/interface/) [35], and the Japanese face images from the Advanced Industrial Science and Technology facial expression database [36]. For each specific type of emotional stimulus, there were five morphed faces that were 10%, 30%, 50%, 70% and 90% in strength. For each intensity point, six faces (three male and three female) were prepared. Therefore, 90 faces (three emotions × five intensities × six faces) were prepared for each condition for this experiment. Although we could not present any stimulus due to the contract with the developer of the facial expression database, readers can find the original images elsewhere [33]. To enhance the credibility of our manipulation, we asked participants to rate their extent of nervousness or anxiety on a six-point Likert scale from 1 = *not at all* to 6 = *very much* for each mask condition. In addition, we prepared a video clip featuring 20 graduate students who were **‘**observing the participant live’. The clip was sourced from a Web meeting system (Zoom), where the students were simply watching their Web camera in a natural manner. This clip was used to induce unrest and anxiety in the participants.

### 2.3. Procedure

Participants were seated approximately 60 cm away from the display. At the beginning of the experiment, they were informed that this was a study about human face recognition and that their faces would be observed by many students from another university throughout the experiment. Later, a video clip of the audience, which was recorded previously through Zoom, was presented as a live image for a while to enhance the reliability of this instruction. The participants were continuously instructed about the rating task, wherein a trial was composed of presenting a fixation (duration was jittered from 0.5 to 2.5 s) and a face stimulus (until button press). They were asked to simply judge the intensity of the faces' emotions from 1 = *weak* to 6 = *strong* using a button press. During the instructions, participants saw every emotional face with every intensity which would be presented in the upcoming task. There were two mask condition sessions (masked or unmasked), each of which consisted of three blocks corresponding to each emotion (i.e. anger, fear and happiness). The orders of block and session were respectively counterbalanced between the participants. Each block had 30 trials, where the order of face image (face identity × facial expression intensity) was pseudo-randomized. At the beginning of each block, the corresponding emotion was displayed to inform participants of the target emotion. The video clip of the audience was presented at the beginning of each block to ensure that the participants were constantly monitored. At the end of each condition, participants were asked to rate the extent of nervousness or anxiety they experienced throughout the condition on a six-point Likert scale. The entire experiment took less than 10 min to complete. Before the experiment, all participants underwent a practice block to familiarize themselves with the task. After the data collection, participants were debriefed.

### 2.4. Analysis

**Figure 1.**
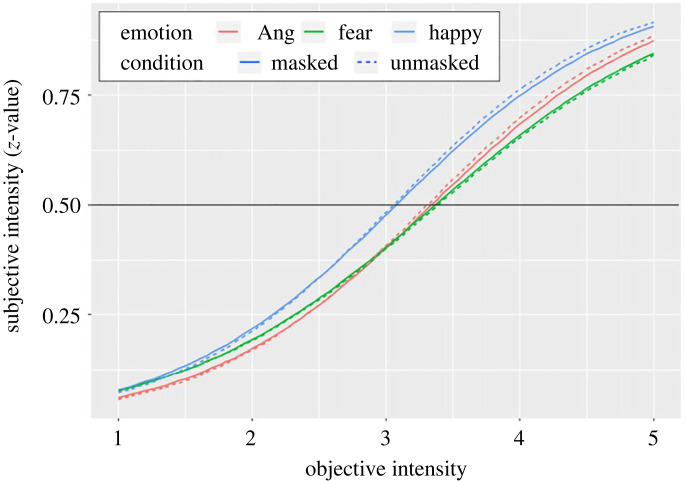
Individual-averaged psychometric function for each emotion and condition. *Note*: point-of-subjective equality was defined as the intersection point of the *y* = 0.5 line and the regression curve.

The R software (https://www.R-project.org/) was used for data analyses and visualizations. To investigate the impact of the condition (i.e. masked or unmasked) on participants’ negative feelings, a pairwise *t*-test was conducted. For the behavioural performance, RT was first analysed to ensure that no other difference such as that of processing time occurred between the two conditions. Emotion processing alteration between the conditions was examined by comparing a 50% threshold point (FTP) of intensity judgement. To calculate them, probit regression was used to model the data with an inverse standard normal distribution of the probability as a linear combination of the predictors. As the data ranged from 1 to 6, we converted them from 0 to 1 via z-transformation. By fitting the probit model by the glm function in R, the intersection point of the 0.5 line and the regression curve were considered as FTP (electronic supplementary material, figures S1 and S2, and figure 1). The FTP represents the value, which is subjectively perceived to be the midmost intensity. Additionally, another traditional index (i.e. just noticeable difference [JND]) was calculated based on the 25% and 75% threshold points of intensities. Those indices were derived and analysed based on their variance (i.e. 2 [condition] × 3 [emotion] factorial design). Where appropriate, the method of Holm was used to adjust the *p*-values in multiple comparisons.

## 3. Results

As none of the participants had noticed that the video clip was not a live broadcast, our deception was successful, and it was assumed that the participants believed they were being monitored during the intensity rating task. In the unmasked condition, the participants rated their negative feelings such as nervousness and anxiety as 3.26 on average (s.d. = 1.38). Regarding the experimental manipulation, wearing a mask significantly reduced participants' negative feelings (2.27 on average [s.d. = 1.00]; *t*_34_ = 6.18, *p* < 0.001, Hedges’ *g* = 0.81).

The condition did not affect processing time (*t*_34_ = 1.15, *p* = 0.255, Hedges' *g* = 0.11). We then analysed whether our manipulation affected emotion intensity recognition. Figure 1 shows the averaged psychometric function fitted with the probit model. Electronic supplementary material, figures show all participants’ results of psychometric function fitted with the probit model in unmasked (electronic supplementary material, figure S1) and masked (electronic supplementary material, figure S2) conditions. An analysis of variance for the FTP indicated the significant effect of emotion (*F*_2,68_ = 18.51, *p* < 0.001, $\eta_{p}^{2}=0.35$), wherein the FTP on the happiness face was significantly lower than that on fear (*p*.adj < 0.001) and anger (*p*.adj = 0.001) faces; no difference was found between fear and anger (*p*.adj = 0.275). The effect of condition (*F*_1,34_ = 0.08, *p* = 0.777, $\eta_{p}^{2}=0.002$) or the interaction (*F*_2,68_ = 0.20, *p* = 0.816, $\eta_{p}^{2}=0.006$) was not significant. The same variance analysis for JND also found a significant effect of emotion (*F*_2,68_ = 19.98, *p* < 0.001, $\eta_{p}^{2}=0.37$) but not of condition (*F*_1,34_ = 0.81, *p* = 0.375, $\eta_{p}^{2}=0.02$) and interaction between emotion and condition (*F*_1,34_ = 0.59, *p* = 0.556, $\eta_{p}^{2}=0.02$).

## 4. Discussion

In the present study, participants were placed in a situation that induced feelings of nervousness and anxiety by informing them that they were being observed by several people and presenting a video clip where many people appeared to look at them. We investigated if wearing a mask eases the negative psychological state caused by such a situation, which would then alter the perceived intensity of facial expressions of emotion. The first hypothesis was clearly supported in this study. Wearing masks can be a way to avoid negative evaluations from others by concealing the face or hindering social interaction with others [3]. This cultural tendency is supported by survey results, indicating that many Japanese believe that wearing a mask enhances facial attractiveness [13]. Although wearing a mask theoretically provides psychological comfort, particularly for the Japanese, no study has directly examined its temporal effects. Here, we quantitatively detected that wearing a mask can reduce the participants' negative feelings with a high effect size (Hedges’ *g* = 0.81). However, given that this observation may be specific to Japanese participants due to their collectivistic culture, future studies should include participants from other cultural backgrounds to further investigate this effect.

This reassuring effect of mask-wearing did not affect the participants' behavioural performance in emotion intensity recognition, as confirmed by two indices (FTP and JND). First, it was anticipated that wearing a mask could alter recognition ability, as demonstrated in previous research [24], indicating the possibility of transient cognitive alterations when wearing masks. Second, the interpretation of less intense (or ambiguous) facial emotions would be distorted when the participants were in a negative mood [32,33]. Thus, under the current experimental settings, participants in a negative mood (i.e. unmasked condition) were supposed to be biased in a negative way in recognizing ambiguous facial emotions. However, we observed that the participants rated every emotional face equally under masked and unmasked conditions. The null result may, first, be due to the small effect of the negative bias in anxiety. Previous studies have reported mixed results in which people with a trait or state of anxiety display greater sensitivity to anger [27,29] and fear [28,30], whereas others reported no effect of anxiety on emotion recognition and interpretation [37,38]. The null result in the present study was caused by a weak relationship between interpretation bias and (at least, state) anxiety or the lower level of anxiety even in the unmasked condition. Second, one could argue that the way of measuring negative feelings in the present study was not sophisticated. Although the index changed due to the condition, we did not use traditional psychological scales such as the State-Trait Anxiety Inventory. We asked participants only once per condition after the condition ended. It is plausible that wearing a mask could reduce a specific aspect of negative feelings. This aspect can be measured using the Likert scale, which is not related to the interpretation bias of facial expressions. Third, we discuss the null result in relation to Freud *et al*.'s study [24]. They found that wearing a mask may impair face recognition performance because it can provide robust somatosensory stimulation similar to enforced face pose or brain stimulation, which reduces recognition. If this is an underlying mechanism for the mask-wearing effect, the blunt recognition of facial expression could be expected in the masked condition, as shown in previous research that used other types of somatosensory stimulation [25,26]. Thus, the current result may not support this hypothesis. Another possibility suggested by Freud *et al*. [24] is that wearing a mask changed the state of the observer concerning other agents, that is, observers wearing a mask might consider that others cannot properly recognize them, which in turn can hinder their face processing abilities (involuntary perspective taking; also known as **‘**altercentric intrusion‘ [39]). Although the context in this explanation seems to fit well with the current experiment in which the participants were informed that they were being monitored by others, their performance was not affected by mask-wearing. The results of this study may provide evidence against the altercentric intrusion hypothesis of the mask-wearing effect. Alternatively, it is possible that altercentric intrusion affects face identity recognition but not emotion intensity perception.

In the present study, we observed a strong effect of mask-wearing on reducing negative feelings and its null effect on changing emotional recognition. Wearing a mask can change an individual's state by physically concealing and psychologically protecting themselves from others. Although the present study obtained no behavioural evidence, wearing a mask might have several effects on the wearer's perception and cognition as reported by Freud *et al*. [24]. Future studies should investigate the possibility of the altercentric intrusion hypothesis on face identity recognition, as well as the potential cultural specificity of the reassuring effect observed in this study, which may be unique to the Japanese collectivistic culture. To avoid confounding cultural effects with the effect of wearing a mask on emotion recognition [40], it is important to carefully consider the cultural influences of participants from non-collectivistic cultures in further research.

## Data Availability

Our data have been made publicly available via the Open Science Framework, which can be accessed at https://osf.io/3gj5d/ The data are provided in electronic supplementary material [41].
